# 818. Therapeutic Drug Monitoring of Triazole Antifungals for Coccidioidal Meningitis

**DOI:** 10.1093/ofid/ofad500.863

**Published:** 2023-11-27

**Authors:** Michelle Fang, Bianca Torres, Lovedip Kooner, Chelsea Dunn, Jessica Redgrave, Rasha Kuran, Arash Heidari, Royce H Johnson

**Affiliations:** Kern Medical, Bakersfield, California; Kern Medical, Bakersfield, California; Valley Fever Institute at Kern Medical, Bakersfield, California; Ross University, Bakersfield, California; Kern Medical, Bakersfield, California; Kern Medical Center, Bakersfield, California; Dignity Health, Bakersfield, CA; Kern Medical Center, Bakersfield, California

## Abstract

**Background:**

Coccidioidal meningitis (CM) is a potentially devastating manifestation of disseminated infection caused by Coccidioides spp., generally requiring lifelong therapy with triazole antifungals (TA). Given the complexity of assessing treatment response in coccidioidomycosis, which can be impacted by host immune response, pharmacokinetics, antifungal pharmacodynamics, and medication adherence, therapeutic drug monitoring (TDM) of TA therapy, which is not currently common practice for most TA, has been proposed to improve clinical evaluation of such variables. Consistent TDM is anticipated to increase the probability of a successful CM treatment outcomes.

**Methods:**

This is a retrospective review of patients with CM who received adjunctive TA TDM at the Valley Fever Institute at Kern Medical from November 2019 to December 2022. Approval and a waiver of consent were obtained from the Institutional Review Board. The combination of a patient and TA was considered a unique therapy regimen, with each TA course evaluated separately for patients who received multiple triazoles over the course of their CM treatment. The primary endpoint was improvement in the cerebrospinal fluid (CSF) score, which was derived from the Mycoses Study Group scoring system for meningeal disease, over the available follow-up period.

**Table 1.**
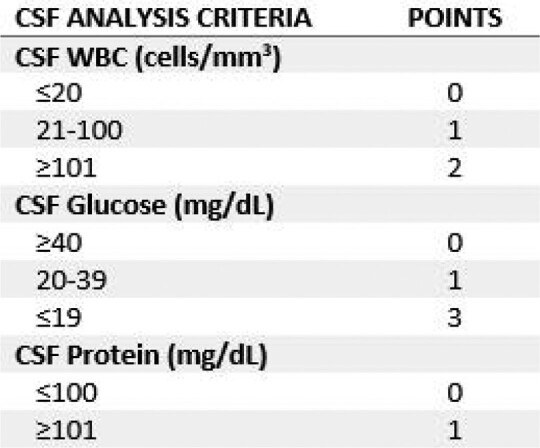
CSF Score (based on the Mycoses Study Group scoring system for meningeal disease)

**Results:**

Twenty-seven TA regimens were found to be eligible for inclusion in this study, with fluconazole comprising the majority of these courses (52%) and the remainder divided between isavuconazole (22%), voriconazole (11%), itraconazole (7%), and posaconazole (7%). Of the serum triazole levels obtained, 34% were considered to be therapeutic, 45% were subtherapeutic, and 21% were supratherapeutic. Greater improvements were noted in CSF scores of TA regimens with the higher serum levels compared to TA regimens resulting in lower serum levels (see figure 1).

**Figure 1.**
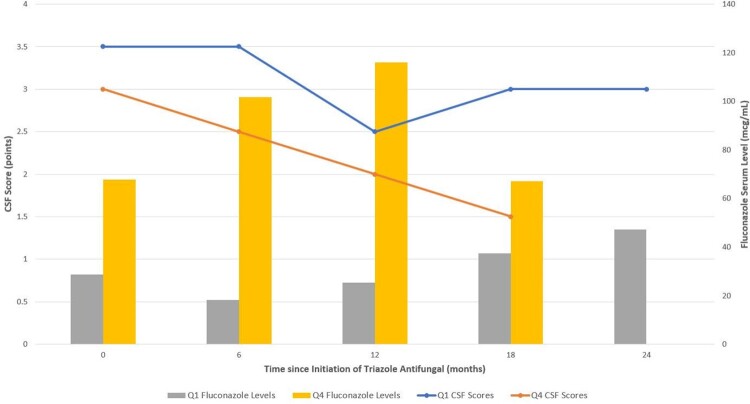
CSF Scores on Fluconazole: Lowest vs. Highest Quartile

CSF scores of fluconazole regimens resulting in the lowest quartile (Q1, n=4) of serum fluconazole levels over 24 months were compared to regimens resulting in the highest quartile (Q4, n=4) of serum levels. A decrease in the CSF score generally represent improvement in meningeal disease.

**Conclusion:**

TA TDM is expected to advantage the care of patients with CM. The possible correlation between higher TA serum levels and improvements in CSF parameters shown in this study emphasizes the potential role of TDM for maximizing TA efficacy in CM, in addition to discernment of medication nonadherence or suboptimal pharmacokinetics.

**Disclosures:**

**All Authors**: No reported disclosures

